# Predicting metabolic preferences through transcriptomics: a data-driven approach to align metabolic signatures with gene expression profiles

**DOI:** 10.1016/j.bbrep.2025.102302

**Published:** 2025-10-22

**Authors:** Lente J.S. Lerink, Marleen J. de Winter, Jaap A. Bakker, Ian P.J. Alwayn, Rutger J. Ploeg, John F. Mulvey, Jan H.N. Lindeman

**Affiliations:** a Department of Surgery & Transplant Centre, Leiden University Medical Centre, Albinusdreef 2, 2333 ZA, Leiden, the Netherlands; b Nuffield Department of Surgical Sciences, University of Oxford, John Radcliffe Hospital, Oxford, OX3 9DU, United Kingdom; c NIHR Oxford Biomedical Research Centre, Oxford University Hospitals Trust, John Radcliffe Hospital, Oxford, OX3 9DU, United Kingdom; d Laboratory Genetic Metabolic Diseases, Amsterdam University Medical Centre, Location AMC, Meibergdreef 9, 1105 AZ, Amsterdam, the Netherlands

**Keywords:** Arteriovenous measurements, Metabolism, Metabolic flux, Substrate preference, Transcriptome profiles, Transcriptomics

## Abstract

Complex organisms such as mammals have a sophisticated metabolic network to meet energy demand under varying conditions. This network, which includes the exchange of metabolites between organs, is absent in *ex vivo* model systems like cell culture or isolated organ perfusion. These systems therefore require external management of metabolic substrates; since failure to meet the specific metabolic requirements will lead to cellular stress, non-physiological behaviour and in turn limited translatability, it should be ensured that model systems exhibit *ex vivo* metabolism that recapitulates *in vivo* processes. To better support but also assess tissue and cell metabolism under *ex vivo* conditions, it is thus crucial to be knowledgeable of their specific *in vivo* metabolic preferences. As *in vivo* organ- and cell-specific metabolic preferences are only partially characterised, a surrogate marker of metabolism is required that can easily be measured in both *in vivo* and *ex vivo* isolated organ or cell culture systems. In an attempt to identify surrogate predictive markers of metabolism that could be easily measured in *ex vivo* model systems, we investigated the extent to which organ-specific metabolite consumption and production patterns (referred to as “metabolic signatures”) from available arteriovenous flux data align with organ-specific metabolic gene expression patterns. Whilst different tissues displayed distinctive patterns in the consumption and production of metabolites, these did not directly correspond to expression of known metabolic genes. These findings are indicative of the complexity of mammalian metabolism.

## Highlights

• Different organs display distinctive metabolite consumption and production patterns.
• Knowledge of metabolic preferences is key to support and assess *ex vivo* models.
• Organs' metabolic gene expression do not align with their arteriovenous flux data.
• Metabolic gene expression patterns cannot be used to predict metabolic preference.
• Control of metabolic flux depends on a complex interplay of many different factors.

## Graphical abstract

**Figure:**
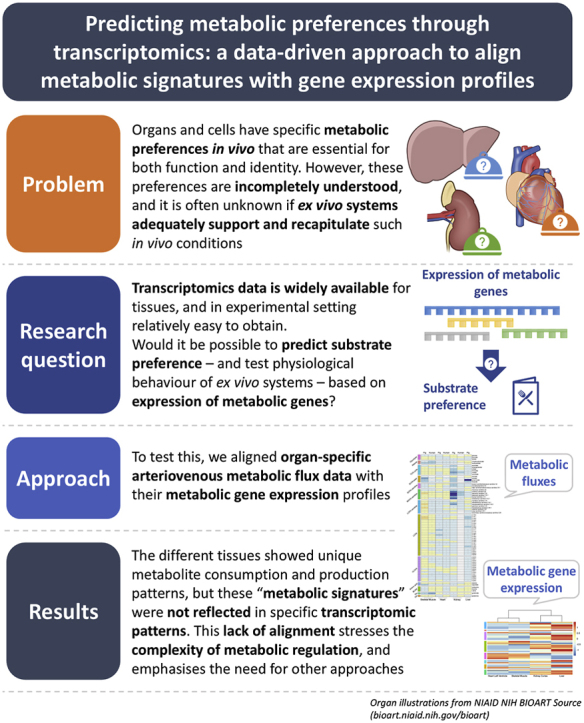

## 1 Introduction

Metabolic competence is a prerequisite to sustain life. To ensure metabolic capacity under widely differing conditions, complex organisms such as mammals are equipped with a dense and partially redundant network of metabolic pathways. This robust network maximises flexibility and adaptability in the face of profound variations in both substrate availability and metabolic demands [1]. Only in pathological conditions or extreme physiologic circumstances (partial) metabolic failure will occur, potentially with devastating consequences [[2], [3], [4], [5]].

Mammalian tissues are specialised to perform specific functions, and consequently organs and cells differ profoundly in their energetic requirements and dynamics: the heart and kidney continuously require vast amounts of energy, whereas the demand of skeletal muscles or leukocytes is minimal under resting conditions but massively increases upon physical activity or cell activation [6]. These differing metabolic needs and dynamics are reflected in broadly varying metabolic preferences [7], which results in metabolism overall being compartmentalised within the organism. Nevertheless, the different compartments within the organism are interdependent: adequate metabolic resilience relies on the exchange of metabolites between different organs. This *in vivo* integrated structure is absent in many experimental model systems, such as cell or organoid culture *in vitro,* or *ex situ* organ perfusion in the context of transplantation. These model systems therefore rely on the exogenous management of metabolite requirements and waste product removal to maintain a balanced metabolic state.

Failure to appropriately manage metabolic support in *in vitro* or *ex situ* research models can result in cellular stress, compromised viability or non-physiologic behaviour, and may impact on cell identity. In fact, it has become increasingly evident that different cell types not only exhibit distinct metabolic signatures, but also that metabolism itself can determine cell fate. For example, metabolic switching is crucial for leukocyte activation [8,9], macrophage polarization [10], cardiac regeneration [11], and also determines fibroblast identity in the process of fibrosis [12]. Based on these observations, one could speculate that these differences in metabolism between pseudo-physiological experimental conditions and the relevant *in vivo* metabolism may contribute to the poor translatability of research findings. Hence, there is an unmet need for tools that allow for the evaluation of metabolic signatures to test whether the metabolic conditions in experimental models sufficiently recapitulate the physiologic *in vivo* conditions. Development of such a tool critically relies on the establishment of metabolic signatures in both situations. However, at present, physiological *in vivo* organ-specific metabolic preferences are only partially characterised: there is a scarcity of organ-specific metabolic flux data [13], which is due in part to the technical challenge of acquiring this data under physiological circumstances. Collecting biopsies and measuring metabolite concentrations for example does not suffice, as static snapshots of concentrations do not capture the dynamic metabolic fluxes between organs and tissues [3]. Therefore, there is an urgent need for strategies that adequately define individual, physiological metabolic signatures.

*In vivo* arteriovenous (AV) sampling across whole organs does enable the capture of metabolic fluxes [13]. This has recently been accomplished in a series of human and porcine studies, providing insight in the diversity of organ-specific patterns of metabolite consumption and production, which we refer to as “organ-specific metabolic signatures”, under (pseudo) physiologic circumstances [3,7,14]. As AV sampling-based data is still limited to whole organs, and the sampling technique itself is naturally not applicable to simplified experimental models, a surrogate marker of tissue metabolism that can be assayed using commonly available analytical techniques to measure the metabolic signature is instead required. This would enable the identification of cell-specific metabolic signatures and would consequently enable researchers to verify that their experimental system is metabolically relevant to clinical translation. Since changes in gene expression have been shown to accompany metabolic switching [[15], [16], [17]], we hypothesised that organ-specific metabolic signatures (i.e. metabolic fluxes) are reflected by organ-specific differences in gene expression. Naturally, these gene expression patterns would not reflect acute metabolic regulation, which is mostly achieved by allosteric regulation and post-translational modification as transcriptomic regulation is a non-acute process [18]. Still, it could potentially predict “baseline” metabolic signatures as observed in a near-stable setting, free of major disturbances. A small number of surrogate markers could then feasibly be measured by qPCR to map metabolic signatures.

In this study, we therefore first defined a signature of metabolic flux in different organs using AV differences in concentration across organs reported previously in the literature. We then used a data-driven approach to explore whether gene expression patterns in a large database covering multiple organs align with these specific metabolic signatures, by combining transcriptomics and prior knowledge about metabolic networks to compare the consensus AV flux signatures with organ-specific transcriptomes of metabolic genes. We finally assessed the feasibility of defining a small set of transcript markers that could practically be used as surrogate markers of metabolic signatures between organs, to be potentially used for cell-specific metabolism as well.

## 2 Methods

### 2.1 Arteriovenous data

**Fig. 1:**
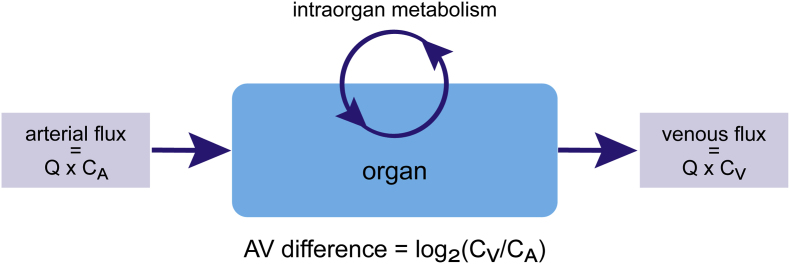
**Arteriovenous measurements.** Arteriovenous (AV) differences are calculated based on the ratio between venous metabolite concentrations (C_V_) and arterial metabolite concentrations (C_A_). Q = flow.

Raw AV data was obtained from the publications by Jang et al., Murashige et al., and Lindeman et al. [3,7,14,19]. Individual venous over arterial ratios were log_2_-transformed, as previously described by Jang et al. (Fig. 1). Subsequently, averages were calculated. Details about the applied experimental procedures and statistical analyses of the included studies can be found in Supplementary Table S1. Of note, skeletal muscle AV ratios are calculated based on femoral vein outflow data, which drains from skeletal muscle, fat, bone and skin. A selection of 79 metabolites of interest was based on previously reported main human metabolic pathways [7,14].

### 2.2 Transcriptomics

#### 2.2.1 Metabolic gene selection

Metabolism-related genes were selected based on the central metabolic pathways' genes listed in HumanGem [20]. Genes classified within the following pathways were included: ‘Glycolysis/Gluconeogenesis’, ‘Alanine, aspartate and glutamate metabolism’, ‘Valine, leucine, and isoleucine metabolism’, ‘Tricarboxylic acid cycle and glyoxylate/dicarboxylate metabolism’, ‘Oxidative phosphorylation’, ‘Carnitine shuttle (cytosolic)’, ‘Carnitine shuttle (mitochondrial)’, ‘Carnitine shuttle (peroxisomal)’ and ‘Fatty acid oxidation’. Twenty-three transporter genes from the – more detailed – MitoCarta 3.0 database completed the gene selection process [21]. This resulted in a total of 387 genes.

#### 2.2.2 Transcriptomics data

Tissue transcriptomics profiles were obtained from the Genotype-Tissue Expression (GTEx) project (version 8, dbGaP Accession phs000424.v8.p2) database as gene read counts [22]. Available data includes: skeletal muscle (n = 803), heart left ventricle (n = 432) and liver data (n = 226). Kidney data was reported as cortex (n = 85) and medulla (n = 4). Samples were filtered for RNA quality (only including RNA with an RNA integrity number >7) and only retaining tissues with >10 samples per tissue, resulting in the following numbers: skeletal muscle n = 728, left ventricle n = 235, kidney cortex n = 13, liver n = 69. Given the limited number of available samples, kidney medulla was excluded.

Read counts were processed using edgeR [23]. Lowly expressed genes were filtered, and normalization was performed using the Trimmed Mean of M-values (TMM) approach to correct for library sizes, under the assumption that the majority of genes are not differentially expressed between tissues [24]. This has been shown to facilitate better comparisons between tissues, where the RNA composition of samples between groups may be very different.

Differential expression was calculated using genewise quasi-likelihood negative binomial generalised log-linear models in edgeR. An absolute log_2_ fold change of 2 and a false discovery rate <0.01 (controlled using the Benjamini and Hochberg method) was considered significant.

Enrichment of metabolic gene sets defined as above were calculated using the fgsea algorithm [25], after the Gene Set Enrichment Analysis method of Subramanian et al. [26].

To define metabolic genes that best distinguish between tissues, we first filtered for genes that were expressed across at least 75 % of the samples within every tissue. A decision tree was then created using rpart within the tidymodels framework. Data were split into test and train sets in a proportion of 1:3, stratified by tissue.

### 2.3 Ethical, technical and resource constraints

This exploratory, hypothesis generating study performs computational analysis based upon previously published, publicly available data. The data with suitable relevance to human metabolism that would be required to directly confirm our findings would require collecting samples from humans *in vivo* and thus presents considerable ethical, technical and resource challenges: as such, the present manuscript does not contain new experimental validation.

## 3 Results

### 3.1 Comparison of arteriovenous data shows distinctive patterns for different tissues in both humans and pigs

**Fig. 2:**
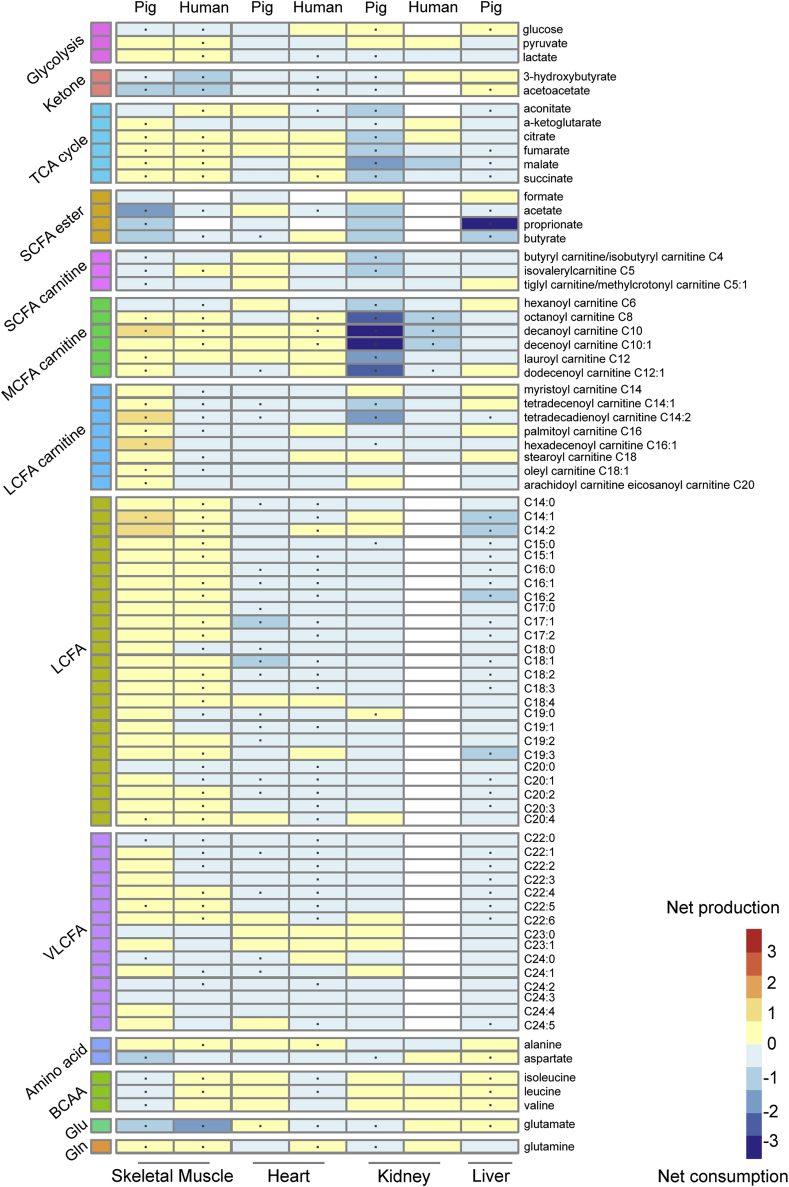
**Overview of the available organ-specific arteriovenous signatures of the main metabolic pathways in skeletal muscle, heart, kidney and liver in pigs and humans.** Jang et al. reported porcine data from skeletal muscle, heart, kidney, and liver [7]. Murashige et al. reported human data from heart and skeletal muscle [14]. Lindeman et al. reported human data from kidney [3]. The average of the log_2_-transformed venous over arterial concentrations are given. Arteriovenous statistically significant differences in metabolites over an organ are marked with a dot. White means no arteriovenous data available. TCA cycle, tricarboxylic acid cycle; SCFA, short chain fatty acid; MCFA, medium chain fatty acid; LCFA, long chain fatty acid; VLCFA, very long chain fatty acid; BCAA, branched chain amino acid; Glu, glutamate; Gln, glutamine.

To summarise the organ-specific uptake and release of metabolites from the major catabolic pathways responsible for the liberation of energy from metabolic substrates (i.e. glycolysis, β-oxidation, tricarboxylic acid (TCA) cycle, branched chain amino acid (BCAA) oxidation, ketone body and ammonium metabolism), a comprehensive heatmap (Fig. 2) of 79 metabolites was created based on the AV data reported by one porcine and two human AV studies [3,7,14]. No data was available on human liver. All sampling was performed after an overnight fast (Supplementary Table S1).

AV-sampling showed that skeletal muscle mostly consumes glucose, ketone bodies, and some short-chain fatty acid (SCFA) carnitines/esters. In turn, medium- and long-chain fatty acids (MCFAs and LCFAs, respectively) and TCA cycle intermediates are released in the femoral vein, which will also contain outflow from subcutaneous adipose tissue, skin and bone. Species differences were observed for LCFA carnitines and amino acids (Fig. 2). Cardiac metabolism is dominated by LCFA and very long chain fatty acid (VLCFA) β-oxidation, as well as ketone metabolism. The human heart also consumes BCAAs. Renal metabolism was characterised by MCFA, ketone body and lactate oxidation, and BCAA and glucose release (gluconeogenesis). Whilst the human kidney produced glutamate and glutamine, these amino acids were consumed by porcine kidneys. Porcine liver metabolism was characterised by the oxidation of LCFA and TCA cycle intermediates, gluconeogenesis and the release of ketone bodies and amino acids (predominantly BCAAs).

### 3.2 Human transcriptomics data

**Fig. 3:**
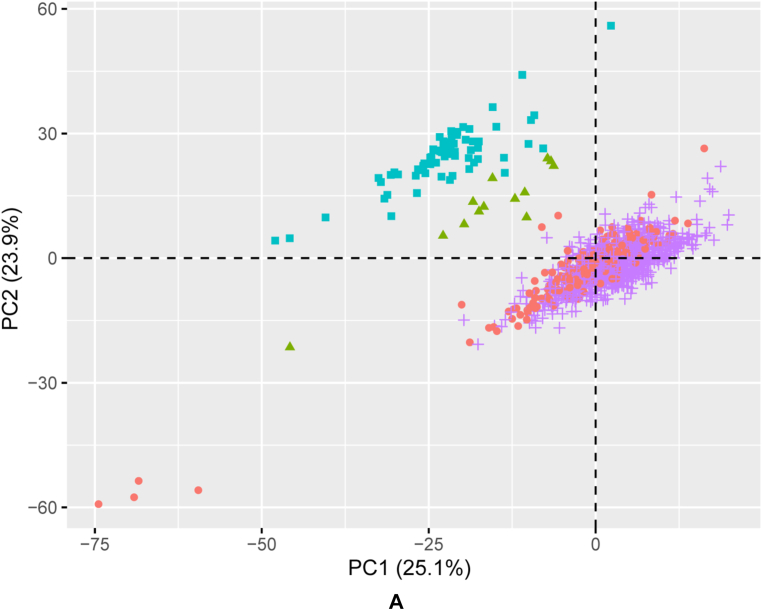

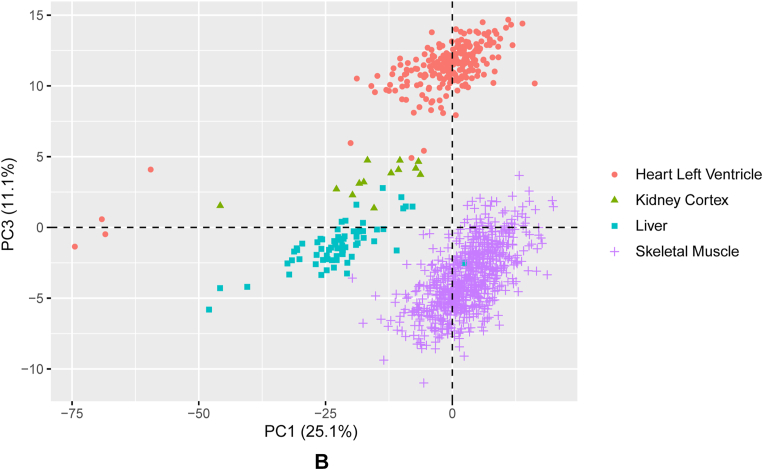

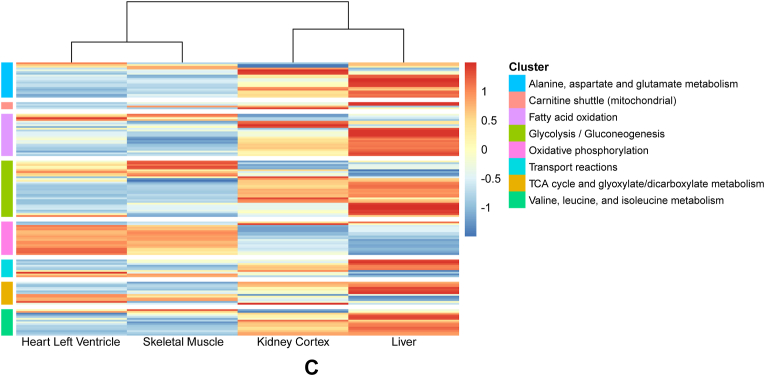
**Principal component analysis and differentialexpression of metabolism-related genes.** (A) Principal component 1 (PC1) vs PC2 and (B) PC1 vs PC3. Orange circles = heart left ventricle (n = 235), green triangles = kidney cortex (n = 13), turquoise squares = liver (n = 69), purple crosses = skeletal muscle (n = 728). (C) Heatmap of metabolism-specific genes that show statistically significant differences in expression between the human heart (left ventricle), skeletal muscle, kidney (cortex) and liver. Gene-wise z-scores are depicted from low (blue) to high (red), showing the average expression across all samples of a tissue.

To investigate the extent to which the metabolic phenotype of each organ relates to its transcriptome, we first defined a selection of 387 genes annotated to be involved in pathways including and adjacent to central carbon metabolism in a comprehensive *in silico* model of human metabolism [20]. Principle component analysis (PCA) demonstrates that differences in the transcriptomic signature of these metabolic genes is captured primarily in the first three components, together capturing 60.1 % of the total variance (Fig. 3A and B, Supplementary Fig. 1A–B). A 5-fold cross validation sensitivity analysis shows that the PCA structure across components 1–3 is stable across all folds (Supplementary Fig. 2). Consistent with the AV metabolic signatures, the liver and kidney show a relatively similar though not overlapping transcriptomic profile. As expected, the heart and skeletal muscle do not overlap with the liver and kidney. The heart and skeletal muscle transcriptomic profiles overlap greatly in the first two principal components (Fig. 3A), in contrast to their clearly distinct AV signatures. Statistical testing showed that many of these metabolism-related genes were found to be differentially regulated between different organs, as summarised in Fig. 3C. Pairwise comparisons between organs demonstrated that the fold changes were generally the smallest between heart and skeletal muscle (Supplementary Material 2).

#### 3.2.1 Enrichment of metabolic pathways between organs

**Fig. 4:**
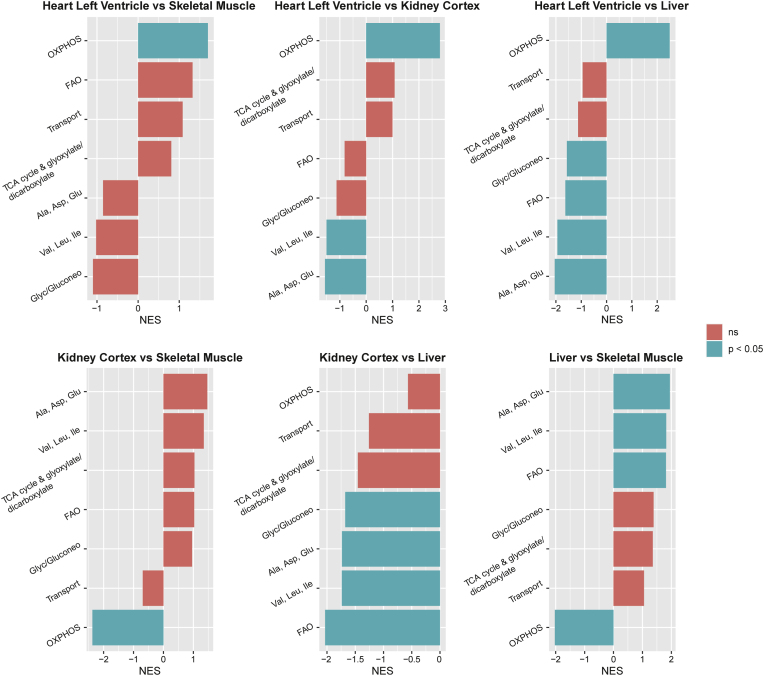
**Gene set enrichment analysis of metabolic genes amongst heart and skeletal muscle; heart and kidney; heart and liver; kidney and skeletal muscle; kidney and liver; liver and skeletal muscle.** NES = normalised enrichment score; Ala, Asp, Glu = alanine, asparagine, and glutamate metabolism; FAO = fatty acid oxidation; Glyc/Gluconeo = glycolysis/gluconeogenesis; OXPHOS = oxidative phosphorylation; Transport = transport reactions; Val, Leu, Ile = valine, leucine, and isoleucine metabolism. Bars are orange if enrichment was non-significant. Bars are turquoise for statistically significant enrichment (adjusted p-value <0.05).

To quantify differences in gene expression at the level of metabolic pathways rather than individual genes, we performed a gene set enrichment analysis (GSEA) using the metabolic pathways defined by the genome scale metabolic model as input (Fig. 4) [20]. The heart shows significant enrichment of oxidative phosphorylation (OXPHOS)-related genes compared to the other organs. Although this is in line with its strong preference for oxidative metabolism, especially through its consumption of LCFAs, the actual enrichment of fatty acid oxidation (FAO) genes is non-significant if compared to skeletal muscle, and even lower compared to liver. Whilst the kidney showed a particularly strong MCFA-favouring metabolic signature, expecting strong enrichment of FAO- and OXPHOS-related genes, its GSEA results demonstrate significant enrichment of alanine, aspartate and glutamate (Ala, Asp, Glu) and valine, leucine and isoleucine (Val, Leu, Ile) metabolism compared to heart, and significantly lower enrichment of OXPHOS compared to heart and skeletal muscle. When compared to liver, the kidney shows no enrichment of pathways. Whilst skeletal muscle was not expected to present enrichment of oxidative metabolic genes, by showing relatively less consumption of fatty acids and TCA intermediates and having substantial glycolytic potential (Cori cycle), a significant enrichment of OXPHOS was found compared to kidney and liver. Interestingly, essentially all metabolic pathway gene clusters are enriched in liver compared to the other organs, apart from OXPHOS, which is enriched in heart and skeletal muscle. This highly enriched but indistinctive metabolic expression pattern does not clearly align with the known human and porcine metabolic fluxes measured by AV sampling (Fig. 1).

#### 3.2.2 Discriminatory metabolic genes: classification into tissue type based on metabolism-related gene expression

Control of flux through metabolic pathways can be regulated by multiple contributing mechanisms, and one reason for poor alignment between the enrichment of pathways and AV concentration differences might be if control was to occur at the level of key intermediate reactions rather than across entire pathways. We therefore also attempted to discriminate between organs using the transcript abundance of only those metabolic genes that were widely expressed across all tissues.

We performed this using decision trees, since they are easily interpretable and select a small subset of features with which to discriminate between tissues. With this classification approach, we were able to achieve excellent performance, with an accuracy of 0.99 assessed on a holdout test set. This distinction could be made based on the expression of pyruvate kinase (pyruvate kinase liver and red cell isoform, *PKLR*), long chain fatty acid CoA ligase 1 (*ACSL1*) and fructose bisphosphatase 2 (*FBP2*) (Supplementary Fig. S1A–C). Increased expression of *PKLR* distinguished kidney and liver from the heart and skeletal muscle. Whilst this may indicate high glycolytic capacity in these tissues, *PKLR* is a known liver- and erythrocyte-specific isozyme of pyruvate kinase, which has a different isozyme for the muscular entities. *ACSL1* expression is higher in the liver than the kidney, corresponding to the liver's role in both the synthesis and degradation of LCFAs and indeed this is consistent with the AV metabolic signature, for which the liver shows a statistically significant consumption of nearly all LCFAs. Fructose bisphosphatase activity is required for gluconeogenesis, and *FBP2* expression discriminates skeletal muscle from the heart, as its expression is higher in skeletal muscle. *FBP2* is canonically described as the skeletal muscle isozyme of fructose bisphosphatase since it is highly expressed in skeletal muscle, in which it contributes to glycogen synthesis [27]. Our data show that both isoforms are however widely expressed across all four tissues profiled here (Supplementary Fig. S1C). This expression of *FBP2* fits with the limited gluconeogenic capacity in the heart, but contrasts with the high gluconeogenic activity (and fructose bisphosphatase activity) of kidney and liver [28], which is likely compensated for by their expressions of *FBP1*, the liver isoform of *FBP2*.

## 4 Discussion

Sustaining energy supply is essential for life; the organism's ability to cope with available nutritional sources is key to survival. Higher organisms such as mammals maintain homeostasis through a complex network of tissues with distinct roles and metabolic identities, tightly connected to cellular identity and function. Collectively, these sustain energy supply even under the most extreme fluctuations in substrate availability.

Such refined, multi-organ metabolic networks are absent in *ex situ* experimental model systems, including cell and organoid culture and isolated organ perfusion. These systems are therefore entirely dependent on exogenous metabolic control, frequently ignorant of the actual physiological preferences. Such application of generalised culturing protocols has been shown to have unintended consequences and limit the external validity of experimental findings [29]. Therefore, there is a need to clarify tissue and cell-specific metabolic preferences. In turn, this will enable successful recapitulation of physiological conditions required for the maintenance of normal functionality and flexibility, cell identity, and consequently the translatability of these systems.

It has been previously suggested that integrating -omics data with the available metabolic flux data may shed light on substrate preference [13]. To test this hypothesis, this study explored whether transcriptomic data is sufficiently discriminatory to reflect organ-specific metabolic signatures. Given the widespread availability of transcriptomic data [30], and the ability to measure the abundance of particular transcripts with common laboratory methods such as qPCR, any predictive value of metabolic transcriptomic data for metabolic preferences could hold great significance for *in vitro* and *ex situ* applications.

### 4.1 AV differences in metabolites reveal organ-specific metabolic signatures

The starting point for this analysis was the identification of physiologic organ-specific metabolic signatures. Tissue concentrations of metabolites represent static snapshots, and do not reflect differences in the flux of metabolites which can vary greatly even whilst tissue concentrations remain unchanged [3]. Disparities in flux are instead most practically quantified through measurements of AV differences. The technically challenging nature of these measurements means that there is a paucity of available AV data, and our first step was to align what was available. Data from skeletal muscle, heart, kidney, and liver were selected due to their distinct metabolic profiles. Since limited human AV data is available and the pig is considered a relevant model for human metabolism, porcine data were also included [31,32]. The resulting AV signatures confirm the metabolic diversity of the different organs, and provides an outline of the clearly distinct, organ-specific metabolic patterns of the main metabolic pathways (Fig. 2). This both retrieved known preferences (such as the heart consuming LCFAs), but our synthesis also provided new insights (such as the strong MCFA preference of kidneys).

Some discrepancies were observed between human and pig AV data (Fig. 2). This was in line with our expectations, as some interspecies differences were expected and we also note that the pigs and the humans differ in their health [33]: whilst the patient groups mostly consist of older individuals (with concomitant comorbidities), the pigs are young and otherwise healthy (Supplementary Table S1). Nevertheless, the overall metabolic signatures of the organs of humans and pigs were similar, and the use of two disparate experimental systems ensured the robustness of the metabolic signatures that we describe.

In short, the AV analysis yielded an insightful overview of organ-specific substrate preferences, which demonstrated clearly discriminative metabolic signatures that aligned with preexisting knowledge.

### 4.2 Expression of metabolism specific genes does not explain organ-specific metabolic signatures

Next, we tested to what extent the clear metabolic signatures might be reflected in differences in the expression of metabolic genes. We therefore performed transcriptomic analyses on a predefined set of 387 key metabolic genes involved in the major metabolic pathways including and adjacent to central carbon metabolism. Different analytical strategies were applied for maximal sensitivity. We showed that whilst there are clear differences in the expression of metabolic genes between organs, both at the level of metabolic pathways and of individual genes, these align poorly with metabolic flux measured by AV sampling.

As the PCA and pathway-level analysis of transcriptomic patterns insufficiently discriminated between the different metabolic signatures of tissues, we further examined the data at the level of individual metabolic genes instead. A method was developed to define genes whose abundances discriminated between the different tissues, which yielded some known differences in tissue metabolic preferences. We propose that this approach might be applicable to obtain insight in the metabolic behaviour of experimental systems, although it should be stressed that our approach is ignorant of organ-specific isozymes and post-translational modifications that should be considered when analysing the data. For example, the high gluconeogenic activity of the liver is not fully captured by the sole expression of *FBP2*.

It also remains difficult to align these findings with metabolic flux data at the level of individual metabolites, since AV sampling measures *net* metabolite production/consumption across an organ rather than the *gross* metabolism that occurs. The kidney is perhaps the best exemplar here, since gradients in oxygen tensions between the cortex and medulla result in large spatial segregation of metabolism: for example with glucose exchange between the gluconeogenic cortex and the glycolytic medulla [34,35]. Due to the low number of samples from the medulla with high quality transcriptomic data in the GTEx database, in this study we included only the transcriptome of the cortex. Despite the contrasting metabolic behaviour of these two regions, it should be emphasised that the cortex is far more metabolically active [36]. Consequently, the contribution of the cortex is expected to be quantitatively dominant. This is shown for example by the kidney's lactate metabolism: lactate is produced in the medulla and oxidised in the cortex, and congruent with the quantitative dominance of the cortex for net flux, lactate is consumed by the kidney on the whole organ level. Metabolism may not necessarily be segregated in space within all organs, but there are also a multitude of examples of metabolite exchange between different cell types. Developments in single cell transcriptomics and proteomics methods will allow this problem to also be tackled from the opposite end [37], explicitly modelling metabolite exchange between different cell types [38].

More targeted methods exist that would be more representative of gross flux, such as the use of either stable isotope or hyperpolarised compounds supplied exogenously which can then be tracked through metabolic networks with either mass spectrometry or NMR-based approaches [[39], [40], [41]]. We regard these methods as helpful as they provide complimentary information to metabolome-wide AV ratios.

### 4.3 Limitations

A limitation of our AV overview is the lack of absolute flux data, as this requires the assessment of regional blood flows which is technically challenging [13]. A more accurate quantitation could be achieved by considering organ-specific blood flow, either through direct measurement or by utilising literature values. For example, if the AV data over an organ with a high blood flow such as the heart or kidney shows a low AV difference, the actual flux of the substrate may still be substantial after multiplying by the high blood flow.

The difficulty in acquiring AV blood samples creates some limitations, with for example the femoral vein being used as a venous sampling site for skeletal muscle, which captures outflow of not only skeletal muscle but also adipose tissue, skin, and bone. In this specific case, we find this assumption reasonable since these are all tissues with relatively low metabolic rates and are thus not expected to contribute significantly. Still, it must be noted that especially subcutaneous adipose tissue may contribute to fatty acid release in the leg [42]. It has previously been pointed out that isotope tracing can be used to overcome this problem by measuring interorgan fluxes, but femoral vein sampling while omitting the outflow from the great saphenous vein could also limit the contamination of other tissues [40,42].

Finally, the transcriptomics data from GTEx, which has been successfully applied in many studies, is derived from tissues from deceased donors that were accepted in organ and tissue transplant programs [22]. Due to activation of multiple biological processes during the process towards (either brain or circulatory) death, it is expected that some genes involved in these processes may be up- or downregulated artefactually. It cannot be ruled out that this also includes some metabolic pathways. Such trade-offs between control over experimental covariates and relevance of the experimental system are common in clinical research. We therefore attempted to mitigate against this by restricting our analysis to only those samples with high quality RNA.

These limitations should be taken into account when interpreting our findings; we emphasise that we aimed to assess the strength of the relationship between organ-specific metabolic signatures and gene expression profiles, and subsequently to generate hypotheses about surrogate markers that are predictive of metabolic preferences. Our results highlight the absence of straightforward, clear links between organ-specific gene expression and metabolic fluxes, but it is essential that further experimental validation with suitable samples be performed before firm conclusions can be drawn.

### 4.4 The complexity of substrate preference: notions for the future

Our observations point towards a more complex interplay of factors that dictate and predict substrate preference beyond gene expression [43,44]. The correlation between mRNA and protein abundance has long been known to be moderate [45], and will undoubtedly account for some of the inconsistency in our efforts to predict metabolic flux. Utilising proteomic rather than transcriptomic data may improve predictions, but large scale efforts to measure the proteome across multiple organs and individuals are currently much less comprehensive than those in transcriptomics [43,46]. The increased though incomplete amount of signal present in the proteome has recently been demonstrated in the heart: Flam and colleagues measured metabolic flux by AV sampling along with transcriptomics and proteomics, and compared differences in metabolism between patients with and without heart failure [47]. Lactate uptake across the heart for example was shown to be increased in patients with heart failure, which could be mapped to differences in abundance of certain glycolytic genes at the level of the protein, but not mRNA. This was not true for all metabolic pathways, however, with ketone consumption related to neither mRNA nor protein abundance. As a first step, future work in a healthy porcine model, combining transcriptomics and proteomics sampling as well as AV sampling with isotope tracing, would enable a multi-omics approach to further capture regulatory layers beyond gene expression within a single model closely reflecting human physiology.

There are a plethora of different mechanisms that have been shown to regulate metabolic flux: post-translational modifications of enzymes, specific activities of enzymes, mitochondrial identity [48,49], as well as overall balances in metabolic networks could all be involved in the development of a specific metabolic preference. For example, whilst the consumption or production of glucose is tightly controlled to maintain blood sugar, for most metabolites there is no (known) comparable mechanism. For at least a subset of metabolites their consumption has been shown to be linearly correlated with their delivery in the blood stream [50], at least when supplying excess levels and comparing within organs. Control by mass action indeed means that metabolic flux will be only partially dependent upon the concentration of the enzymes responsible, and for these parts of metabolic networks therefore predictability from either transcript or protein abundance will always be low. Furthermore, substrate or product inhibition of enzymes also affect the velocity of the reaction.

Besides this, the differences in organ-specific metabolic rates should be considered, which may influence metabolic behaviour. For example, the heart's constant high ATP demand suggests limited engagement in anabolic activities such as gluconeogenesis, favouring direct nutrient utilisation, and it could be hypothesised that the principal reliance on LCFA oxidation prevents recruitment of glycolytic routes. In contrast, skeletal muscle, with intermittent rest periods, has the opportunity for anabolic processes, and relies on recruitment of glycolysis in situations of high energy demand. Finally, for complex organisms, awareness of inter- and intraspecies differences in microbiome is of importance, as this is known to contribute to metabolic differences as well [51].

## 5 Conclusions

This study shows that the alignment of metabolic flux data from AV differences results in an overview of metabolic preferences of skeletal muscle, heart, kidney and liver. Their respective different metabolic signatures cannot be predicted solely on the basis of organ-specific metabolic gene expression. Our findings emphasise that control of flux through metabolic networks is dictated by a complex interplay of many different factors and in practice, it might be necessary to measure metabolite flux directly rather than by using surrogates such as transcript abundance.

## CRediT authorship contribution statement

**Lente J.S. Lerink:** Writing – review & editing, Writing – original draft, Visualization, Project administration, Methodology, Investigation, Formal analysis, Conceptualization. **Marleen J. de Winter:** Writing – review & editing, Writing – original draft, Investigation. **Jaap A. Bakker:** Writing – review & editing, Conceptualization. **Ian P.J. Alwayn:** Writing – review & editing, Conceptualization. **Rutger J. Ploeg:** Writing – review & editing, Conceptualization. **John F. Mulvey:** Writing – review & editing, Writing – original draft, Visualization, Software, Methodology, Investigation, Formal analysis, Conceptualization. **Jan H.N. Lindeman:** Writing – review & editing, Writing – original draft, Methodology, Investigation, Conceptualization.

## Data availability statement

The arteriovenous and transcriptomic datasets analysed in this study are publicly available from previously published studies and publicly accessible repositories, as detailed in the Methods section. The code to reproduce our analysis is accessible via Zenodo: DOI 10.5281/zenodo.16895454. For instructions, visit: https://github.com/john-mulvey/heterogeneity_of_tissue_metabolism.

## Declaration of competing interest

The authors declare that they have no known competing financial interests or personal relationships that could have appeared to influence the work reported in this paper.

## Data Availability

Data will be made available on request.
