# Supplementary material for: Predicting metabolic preferences through transcriptomics: a data-driven approach to align metabolic signatures with gene expression profiles
Source: Biochem Biophys Rep. 2025 Oct 22;44:102302. doi: 10.1016/j.bbrep.2025.102302 (PMC12580596; doi:10.1016/j.bbrep.2025.102302)
Supplement: Multimedia component 1 [file mmc1.docx]

Supplementary Data 1

**Supplementary Table S1. Overview of the methods of the included studies for the arteriovenous (AV) data depicted in Figure 2.**

|  | **Jang et al. 2019** | **Murashige et al. 2020** | **Lindeman et al. 2020** |
| --- | --- | --- | --- |
| **Organism** | Pig | Human | Human |
| **Age (mean ± SD)** | 5 months | 63.95 ± 12.32 yrs | 58.5 ± 5.00 yrs |
| **n** | 5 | 87 | 8 |
| **Fasted** | Overnight | Overnight | Overnight |
| **Notes on population** | Yorkshire pigs, ~50 kg | Patients with preserved ejection fraction undergoing elective radiofrequency catheter ablation for treatment of atrial fibrillation or ventricular tachycardia | Patients undergoing a living kidney graft transplantation, AV measurements during timeframe 10-30 min post-reperfusion |
| **Arteriovenous data** | | | |
| **Heart** | Coronary sinus/carotid artery | Coronary sinus/radial artery | - |
| **Skeletal muscle** | Femoral vein/carotid artery | Femoral vein/radial artery | - |
| **Liver** | Hepatic vein/weighted average of portal vein (78%) and hepatic artery (22%) | - | - |
| **Kidney** | Renal vein/carotid artery | - | Renal vein/arterial line |
| **Statistical analysis** | Two-tailed unpaired student’s t-test for p-values AV differences. Multiple testing correction: Benjamani-Hochberg method, false-discovery rate cut off of 0.05 | One-sample Wilcoxon test for p-values AV differences. Multiple testing correction: Benjamani-Hochberg method, false-discovery rate cut off of 0.05 | Linear mixed model for AV differences. No multiple testing correction – observations are part of theoretical networks |

**A
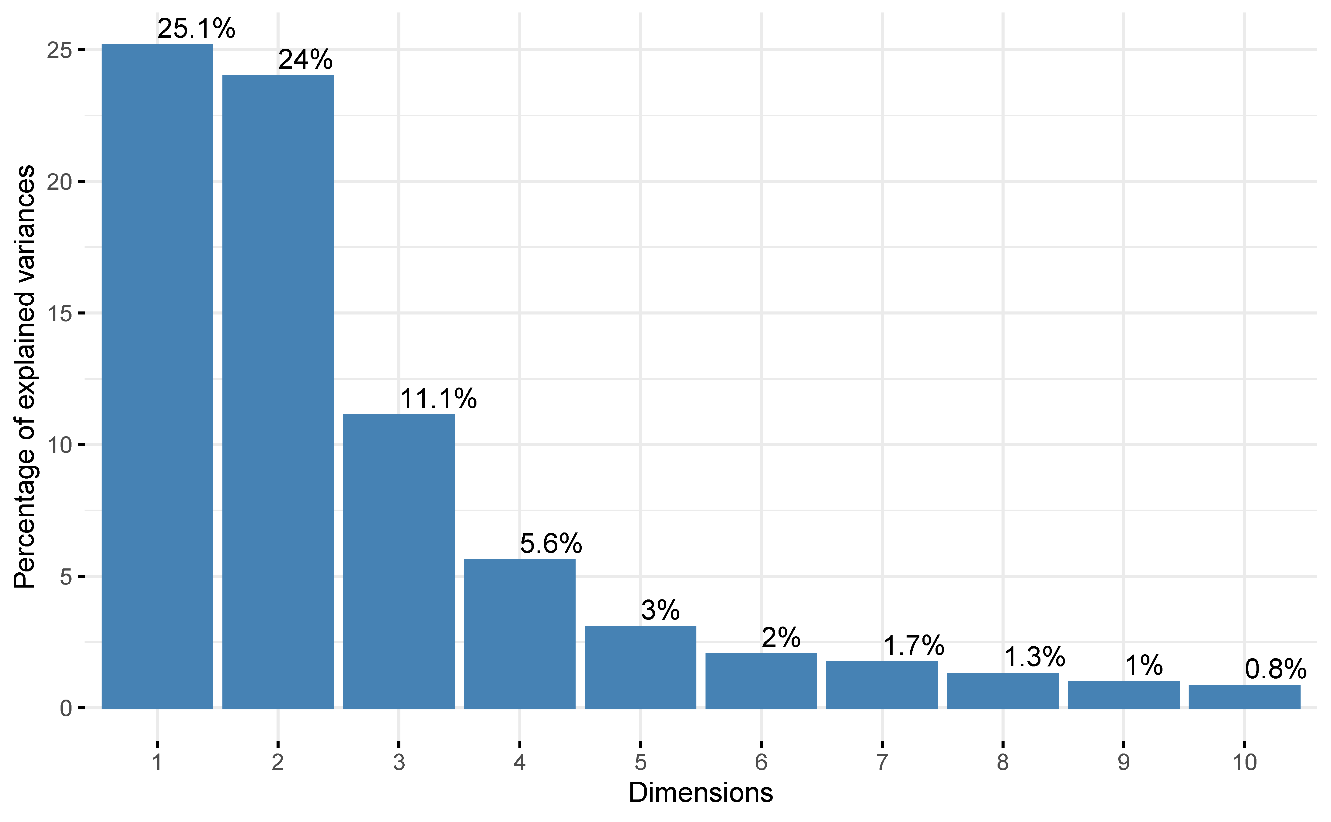
**

**B**
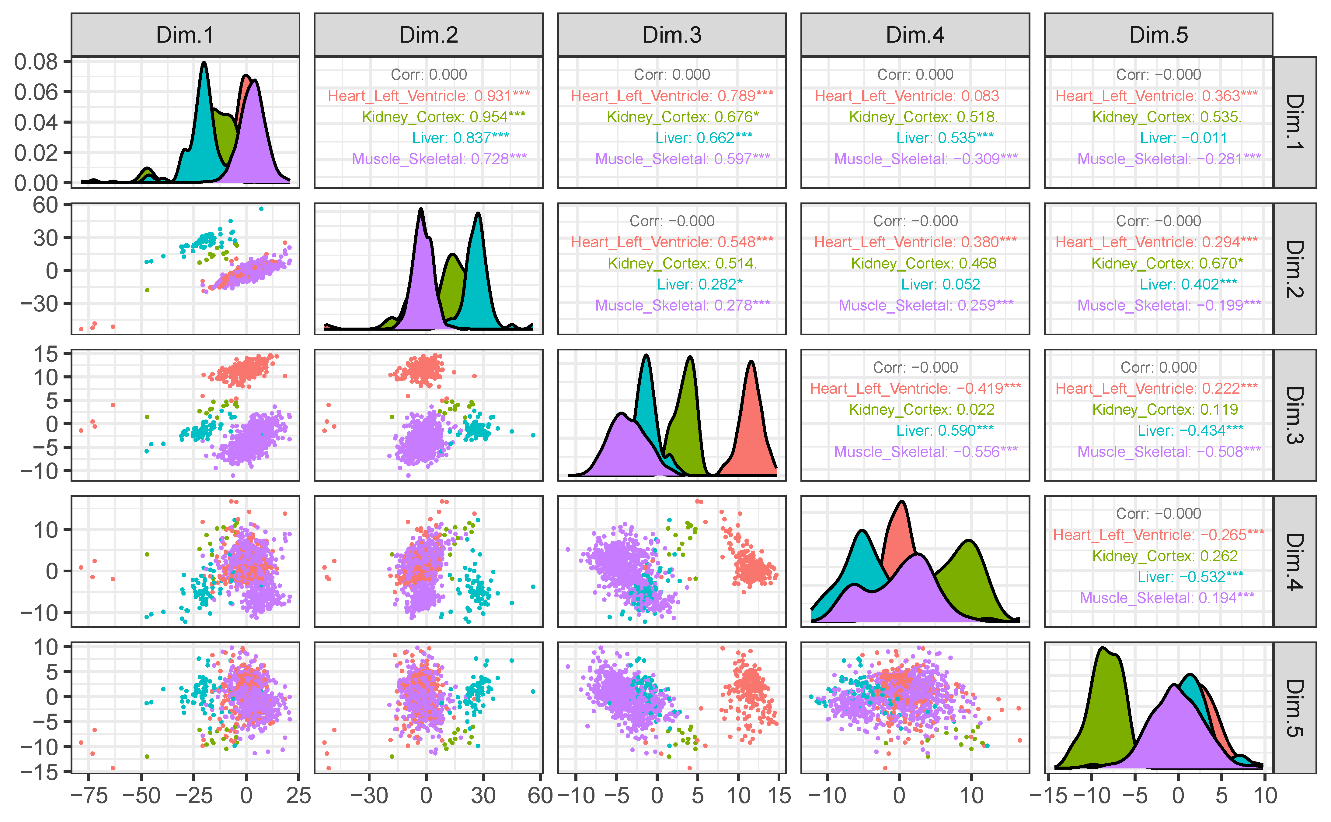


**Supplementary Figure S1. Variance explained by the top-10 principal components and pairwise comparisons of the top-5 principal components from the principal components analysis.** (A) Scree plot showing the percentage of explained variances covered by the top-10 principal components, (B) Pairwise scatterplots across the top-5 principal components. For each tissue as indicated, the Pearson correlation between the relevant principal components is shown above the diagonal, where asterisks indicate the significance of the correlation. Orange = heart left ventricle n = 235, green = kidney cortex n = 13, turquoise = liver n = 69, purple = skeletal muscle n = 728.

**A
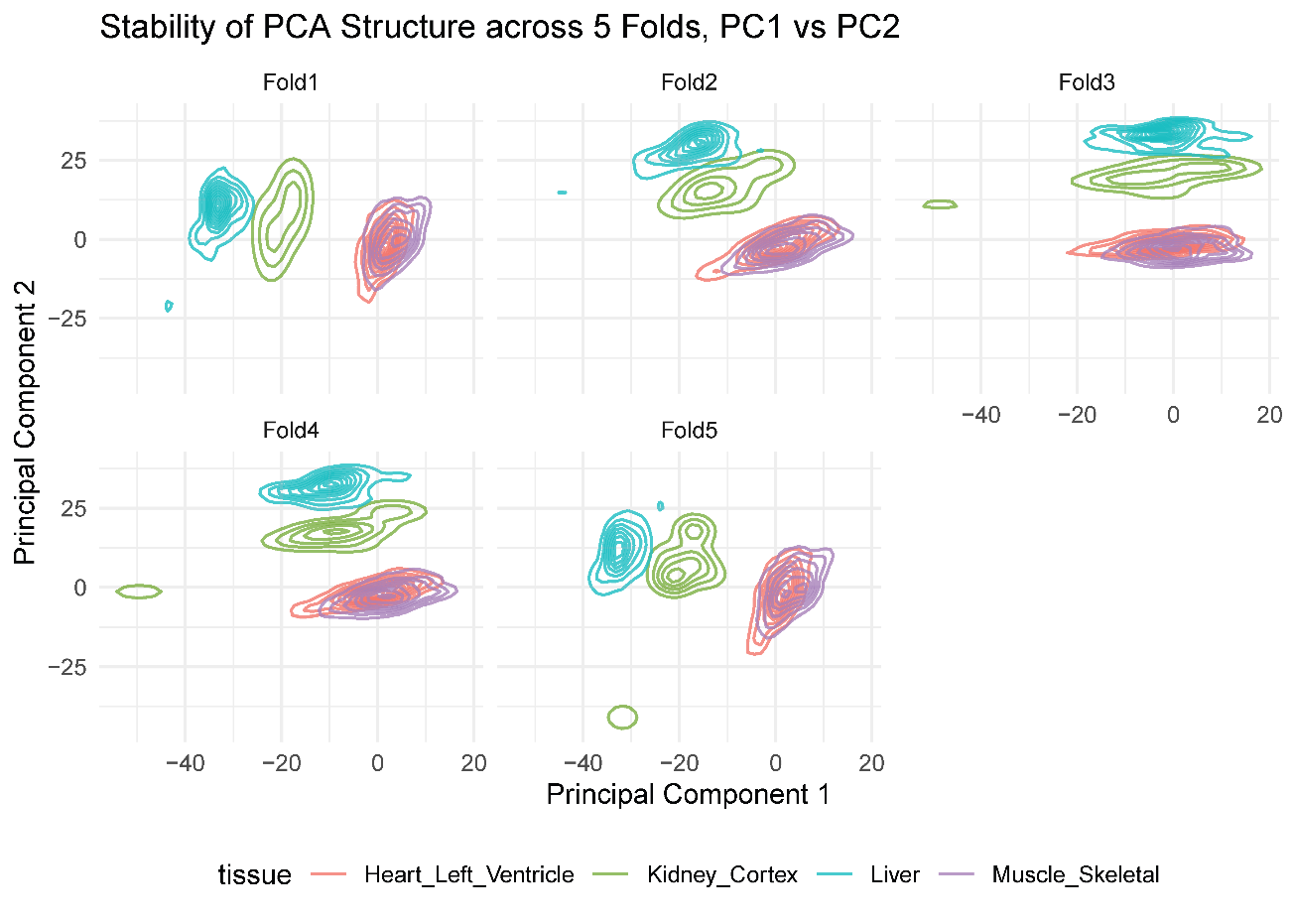
**

**B
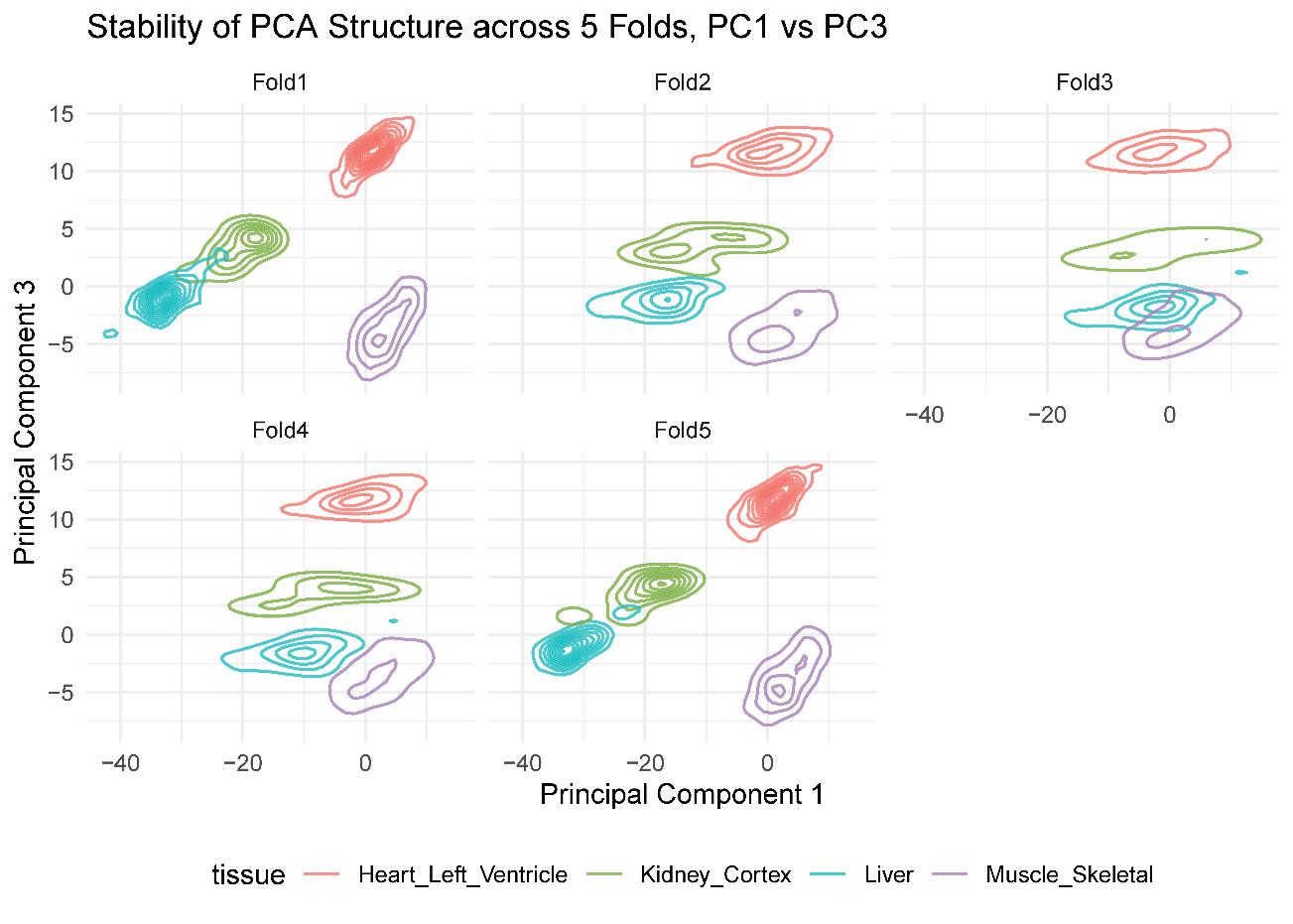
**

**Supplementary Figure S2. Sensitivity analysis of the principal component analysis.** Five-fold cross validation (stratified by tissue of origin) was performed in order to assess the sensitivity of the principal component analysis to the particular samples included in the analysis. (A) PCA of each of the 5 folds, plotting sample density per tissue type on component 1 (PC1) vs PC2, (B) PCA of each of the 5 folds, plotting sample density per tissue type on PC1 vs PC3. Across components 1-3 the gross structure between tissues is insensitive to the subset of datapoints included. Orange = heart left ventricle n = 235, green = kidney cortex n = 13, turquoise = liver n = 69, purple = skeletal muscle n = 728.

**A

**

**B

**

**C

**

**Supplementary Figure S3. Violin plots depicting the relative expression of the specified set of genes that are distinctive for the different organs.** (A) pyruvate kinase liver and red cell (PLKR), (B) long chain fatty acid CoA ligase 1 (ACSL1), and (C) fructose bisphosphatase 2 (FBP2). Expression values are given as log(Transcripts Per Million) of the Trimmed Mean of M-values-normalised expression data.
